# Sustainable power management in light electric vehicles with hybrid energy storage and machine learning control

**DOI:** 10.1038/s41598-024-55988-5

**Published:** 2024-03-07

**Authors:** R. Punyavathi, A. Pandian, Arvind R. Singh, Mohit Bajaj, Milkias Berhanu Tuka, Vojtech Blazek

**Affiliations:** 1 Department of EEE, Koneru Lakshmaiah Education Foundation, Vaddeswaram, Andhra Pradesh 522302 India; 2 Department of Electrical Engineering, School of Physics and Electronic Engineering, Hanjiang Normal University, Hubei Shiyan, 442000 People’s Republic of China; 3 Department of Electrical Engineering, Graphic Era (Deemed to Be University), Dehradun, 248002 India; 4 Hourani Center for Applied Scientific Research, Al-Ahliyya Amman University, Amman, Jordan; 5 Graphic Era Hill University, Dehradun, 248002 India; 6 Applied Science Research Center, Applied Science Private University, Amman, 11937 Jordan; 7 Department of Electrical and Computer Engineering, College of Engineering, Addis Ababa Science and Technology University, Addis Ababa, Ethiopia; 8 ENET Centre, VSB—Technical University of Ostrava, 708 00 Ostrava, Czech Republic

**Keywords:** Solar electric vehicle, Sustainable power management, Light electric vehicles, Hybrid energy storage solution, Supercapacitors, PV-battery interface, SRM EV drive, Machine learning, Energy science and technology, Engineering, Mathematics and computing

## Abstract

This paper presents a cutting-edge Sustainable Power Management System for Light Electric Vehicles (LEVs) using a Hybrid Energy Storage Solution (HESS) integrated with Machine Learning (ML)-enhanced control. The system's central feature is its ability to harness renewable energy sources, such as Photovoltaic (PV) panels and supercapacitors, which overcome traditional battery-dependent constraints. The proposed control algorithm orchestrates power sharing among the battery, supercapacitor, and PV sources, optimizing the utilization of available renewable energy and ensuring stringent voltage regulation of the DC bus. Notably, the ML-based control ensures precise torque and speed regulation, resulting in significantly reduced torque ripple and transient response times. In practical terms, the system maintains the DC bus voltage within a mere 2.7% deviation from the nominal value under various operating conditions, a substantial improvement over existing systems. Furthermore, the supercapacitor excels at managing rapid variations in load power, while the battery adjusts smoothly to meet the demands. Simulation results confirm the system's robust performance. The HESS effectively maintains voltage stability, even under the most challenging conditions. Additionally, its torque response is exceptionally robust, with negligible steady-state torque ripple and fast transient response times. The system also handles speed reversal commands efficiently, a vital feature for real-world applications. By showcasing these capabilities, the paper lays the groundwork for a more sustainable and efficient future for LEVs, suggesting pathways for scalable and advanced electric mobility solutions.

## Introduction

The rising demand for environmentally sustainable transportation has led to a surge in the adoption of electric vehicles (EVs), particularly in urban environments^1^. This trend is underpinned by advancements in battery technology, which have made EVs more viable and cost-effective^2,3^. However, while batteries are integral to EVs, their limitations in terms of energy density and charging times can be restrictive, especially in applications where frequent start-stop or acceleration and deceleration cycles are common, such as in light electric vehicles (LEVs)^4^. This limitation has prompted research into alternative energy storage solutions that can complement batteries, particularly in LEVs. One such solution is the integration of supercapacitors, known for their high power density and rapid charge–discharge characteristics^5,6^. The combination of batteries and supercapacitors (known as a hybrid energy storage system or HESS) offers the potential to address the power and energy density requirements of LEVs more effectively, improving their performance and extending their range^7^. Moreover, the integration of renewable energy sources like photovoltaic (PV) panels offers an added sustainability dimension to LEVs. PV panels can harness solar energy to charge the energy storage system, reducing the reliance on grid electricity and further enhancing the environmental benefits of LEVs^8,9^. Compact and efficient power trains are essential for light motor solar electric vehicles, significantly impacting their productivity. The size of the power electronic interface plays a pivotal role in determining the design of lighter power trains for photovoltaic (PV) assisted electric vehicles^10,11^. This study aims to investigate two critical aspects of the power electronic interface: the development of a lighter hybrid PV, battery, and supercapacitor power supply (HPS) and a lighter SRM converter for electric vehicle (EV) power trains^12,13^. Additionally, this study delves into the realm of efficient and coordinated control through machine learning, presenting a means of achieving an efficient drive system^14,15^. Various hybrid power systems, including PV, battery, fuel cell, and others^16^, have been extensively reviewed for their application in light solar EVs. To interface multiple sources to the DC bus, multi-input non-isolated converters have been proposed^17,18^. These converters, integrated with fuzzy logic control, can dynamically determine the instantaneous power share among the various sources, contributing to an optimized power management scheme^19,20^. Furthermore, a novel battery-super capacitor energy storage system^21^ has been developed with a joint control strategy for average and ripple current sharing. This system addresses the dynamic energy storage and discharge requirements of light EVs, contributing to improved performance and efficiency. The development of a light and efficient power electronic interface, alongside intelligent and coordinated control strategies, is pivotal for the widespread adoption and success of PV-assisted light electric vehicles in the future^22,23^. In the domain of power electronics, bi-directional power flow has emerged as a vital feature for facilitating regeneration during braking in light motor solar electric vehicles. For this purpose, interfacing converters have been equipped with bi-directional power flow capabilities, enabling the integration of hybrid power from photovoltaic (PV) and battery sources^24^. Furthermore, an enhanced DC bus regulation has been achieved through the development of an additional stage for battery interfacing using three-level converters. This advancement not only reduces the size and stress of components but also facilitates battery charging while ensuring power factor correction during the charging process from the utility grid^25,26^. The single-stage integration of hybrid power eliminates the need for a maximum power point converter at the PV interface, thereby simplifying the topology^27^. Efforts have also been made towards optimizing the sizes of power sources according to specific applications, improving bi-directional power conversion capability, integrating various functions into a single converter, conducting thermal stability analysis, and integrating auxiliary functions into the interface converter^28–35^. However, these advanced topologies, with their merits of multiple source interfaces, have also led to complex interfaces and an increased number of power converters and associated filter components^36,37^.

In the realm of control strategies, various models, including model-based, predictive control, and heuristic approaches, have been developed for efficient power sharing and rapid dynamic responses in the switched reluctance motor (SRM) drive^38–40^. These approaches encompass heuristic methods such as genetic algorithms^38^, energy scheduling based on predictive demand^41^, and hierarchical power allocation predicated on the C-rate of the battery and PV power availability^42,43^, aimed at facilitating current sharing among the available sources in a hybrid power supply^44^. Genetic algorithms, for instance, provide an approach to optimizing the current distribution among the different power sources to meet the load requirements, enhancing the overall efficiency and responsiveness of the system^38^. Other strategies include model predictive current reference generation, which leverages mathematical models to predict future current demands^45^, driving cycle-based power demand estimation and sharing function determination, which use historical data on driving patterns to estimate future power requirements^46^, and anticipatory demand control, which anticipates future demand changes based on a range of inputs, such as weather conditions and driver behavior^47^. Recent advancements in control coordination have introduced machine learning techniques such as artificial neural network (ANN) based deep reinforcement learning^48^, ANN for system dynamics estimation^49^, and virtual energy hubs^50,51^, which are being utilized for the control of power conversion. ANN-based methods have the ability to learn from data and adjust control strategies accordingly, making them highly adaptable to varying conditions and requirements. Notable innovations in SRM current control involve the use of fuzzy logic to determine torque reference and instantaneous current^52^, supervised learning for torque ripple minimization^53^, and modified output voltage shape with multi-level converters for improved torque response . Fuzzy logic control provides a more intuitive way to control torque and current in an SRM, whereas supervised learning methods can be used to fine-tune control parameters based on real-world data, enhancing overall efficiency and performance. Modified output voltage shapes with multi-level converters, meanwhile, can provide better torque response and smoother operation by adjusting the voltage waveform to match the motor's requirements^54^. Additionally, dead-beat control based on the motor model has been employed to minimize torque ripple^55^, and online learning techniques have been used for torque sharing function to enhance steady-state and dynamic drive response. Dead-beat control, for instance, uses a motor model to predict future torque demands and adjust control parameters accordingly, while online learning techniques enable the control system to adapt and improve its performance over time based on real-time feedback.

The research problem addressed in this paper is the optimization of power management in light electric vehicles (LEVs) through the integration of a hybrid energy storage solution (HESS) and machine learning-enhanced control. Specifically, the focus is on achieving optimal power flow between batteries, supercapacitors, and photovoltaic (PV) panels to improve vehicle performance, extend battery life, and increase the sustainability of LEVs. Traditionally, LEVs have relied solely on batteries for energy storage, which can be limiting due to their energy density, charging times, and life cycle limitations. The integration of supercapacitors offers a solution to these limitations, as supercapacitors have high power density, rapid charge–discharge characteristics, and longer lifespans compared to batteries. Additionally, the use of renewable energy sources such as PV panels further enhances the sustainability of LEVs by reducing the reliance on grid electricity. However, effectively managing the power flow between batteries, supercapacitors, and PV panels is challenging, especially in dynamic and nonlinear LEV systems. Traditional control strategies may struggle to optimize power flow in real-time, resulting in suboptimal performance and reduced battery life.

To address this challenge, this paper proposes a novel control strategy that integrates a HESS comprising batteries, supercapacitors, and PV panels with machine learning algorithms. By leveraging ML's ability to learn and adapt to complex and changing systems, the proposed control strategy aims to optimize power flow in real-time, ensuring optimal performance and efficiency.

The key contributions of this paper include:

- The development and implementation of a novel control strategy for LEVs that integrates a HESS with machine learning algorithms.
- The demonstration of the feasibility and effectiveness of the proposed control strategy in a real-world LEV application, showcasing its ability to optimize power flow, enhance vehicle performance, and extend battery life.
- The validation of the proposed control strategy's ability to increase the sustainability of LEVs by reducing their reliance on grid electricity and enhancing their overall efficiency.

The findings of this research have significant implications for the design and operation of LEVs, as they offer a more sustainable and efficient alternative to traditional battery-powered vehicles. Additionally, the proposed control strategy has the potential to be applied to other types of electric vehicles, as well as other energy storage and renewable energy systems, further expanding its impact on the field of sustainable transportation.

The paper is organized as follows: In Section "System modelling", we detail the hybrid energy storage solution (HESS), outlining its integration of batteries, supercapacitors, and photovoltaic panels. In this section, we also present the mathematical models that describe the dynamics and behavior of the proposed drive system. Section "Controller modelling" covers the control structure for the proposed converters, including the machine learning-enhanced control strategy designed to optimize power flow between the various energy storage elements. In Section "Simulation results and performance evaluation", we share the simulation setup, including performance metrics and results from the validation of the proposed system. We discuss improvements in power efficiency, battery life, and overall LEV performance. Finally, in Section "Conclusion and future research directions", we offer a summary of the key findings and contributions of the study, along with implications for future research and development in sustainable transportation and energy management.

## System modelling

**Figure 1:**
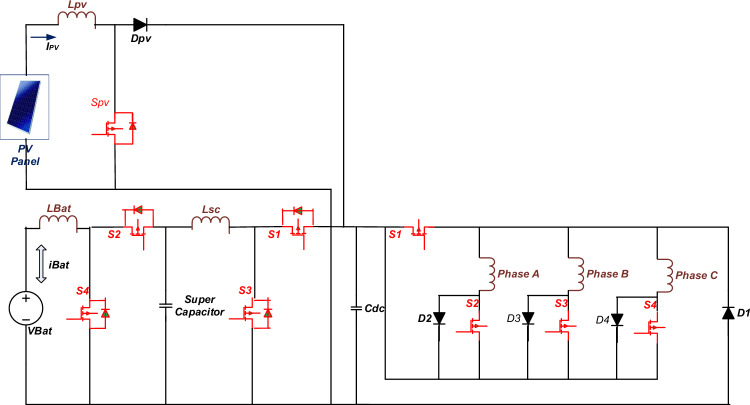
Schematic of HPS-fed SRM drive for light electric vehicle.

With the objective of reducing the size of the power conversion interface for electric vehicle drive firstly, a Hybrid Power Supply (HPS), which integrates battery power into a DC bus in two cascaded stages and PV power in one stage is developed as shown in Fig. 1^56,57^. The power converter associated with PV source is a unidirectional converter which feeds PV power into DC bus through boost converter^58,59^. The objective of control of the boost converter is necessarily maximum power absorption and transfer to the DC bus. The power converters associated with Battery and Supercapacitor is bi-directional converters. Switch S_1_ facilitates the buck mode of operation for transferring power from DC bus to battery while switch S_3_ facilitates the transfer of power from the Battery to the DC bus. Similar operation is achieved for supercapacitor with switches S_2_ and S_4,_ respectively. *L*_*Bat*_ and *L*_*sc*_ serve as filter inductors for the transfer of power. The battery feeds the supercapacitor bus in the first stage, which feeds the DC bus in the second stage. The proposed topology has two advantages. First, the size of the inductor between the battery and supercapacitor interface, *L*_*Bat*_, is reduced compared with conventional topology for the same allowable current ripple. Second, the voltage stress on the power switches at the battery-supercapacitor interface is reduced as compared to conventional topology. Secondly, the number of power switches in the SRM power converter is also reduce to four by maintaining one switch common in commutation of each phase as shown in Fig. 1. The operation of this converter is like an asymmetric bridge converter with the duty cycle of common switch is thrice to that of other switches. Switch G_1_ commutates in common to all three phases which is connected to high side of HPS. Switches G_2_, G_3_, and G_4_ commutate, respectively for each phase connected to the low side of HPS. The 6/4 pole SRM is controlled through direct torque control scheme with reference generated through machine learning-based torque estimation, as seen from Fig. 1. Space vector modulation is utilized for the current control of the drive.

### Mathematical model of the system

#### Hybrid power supply dynamics

The differential equations governing the switching of PV converter are given in (1) and (2), where *i*_*PV*_ and *V*_*PV*_ are the instantaneous current and voltage of PV source, *d*_*PV*_ is the duty cycle of converter, *V*_*Bus*_ is the DC bus voltage, *L*_*PV*_ is the filter inductor in interface, *A* is the material constant of PV array.

1 $$\frac{{di}_{PV}}{dt}=\frac{{V}_{PV}-(1-{d}_{PV}){V}_{Bus}}{{L}_{PV}}$$

2 $${i}_{PV}={i}_{SC}({e}^{{AV}_{PV}}-1)$$

Now, the maximum power condition is achieved at the instant where.

3 $$\frac{{dP}_{PV}}{{di}_{PV}}=0\mathrm{ \,and \,}\frac{{dP}_{PV}}{{dV}_{PV}}=0$$

4 $$\mathrm{Considering \,}PPV=VPV.iPV, \frac{{dP}_{PV}}{{di}_{PV}}=\frac{d}{dt}\left({V}_{PV}{i}_{PV}\right)= {V}_{PV}+{i}_{PV}\frac{d}{{di}_{PV}}{V}_{PV}$$

Now at maximum power point, according to Eq. (3) $\frac{{dP}_{PV}}{{di}_{PV}}=0$ which implies

5 $${V}_{PV}+{i}_{PV}\frac{d}{{di}_{PV}}{V}_{PV}=0 \,{\text{implies}} \frac{{dV}_{PV}}{{di}_{PV}}+ \frac{{V}_{PV}}{{i}_{PV}}=0$$

Discretizing Eqs. (1) and (5), we get

6 $$\frac{{i}_{PV} \left(k+1\right)-{i}_{PV}(k)}{{t}_{s}}=\frac{{V}_{PV} (k)-(1-{d}_{PV} (k+1)){V}_{Bus}(k)}{{L}_{PV}}$$

7 $$\frac{{V}_{PV} \left(k+1\right)-{V}_{PV}(k)}{{i}_{PV} \left(k+1\right)-{i}_{PV} \left(k\right))}+ \frac{{V}_{PV}\left(k\right)}{{i}_{PV} (k)}=0$$

where ${t}_{s}$ is the sampling time and is the reciprocal of switching frequency.

${d}_{PV} (k+1)$ is thus calculated from (6) with sampled values satisfying Eq. (7) which corresponds to maximum power point operation.

The differential equation governing the switching of supercapacitor interface converter is given in (8) , where *i*_*sc*_ and *V*_*sc*_ are the instantaneous current and voltage of Battery, *d*_*1*_ is the duty cycle of battery interface converter, *V*_*Bus*_ is the DC bus voltage, *L*_*sc*_ is the filter inductor in interface.

8 $${L}_{sc}\frac{d{i}_{sc}(t)}{dt}= {V}_{sc}\left(t\right)- {d}_{1}(t){V}_{Bus}(t)$$

Discretizing the differential equation,

9 $${L}_{sc}\frac{{i}_{sc}\left(k+1\right)-{i}_{sc}\left(k\right)}{{t}_{s}}= {V}_{sc}\left(k\right)- {d}_{1}(k+1){V}_{Bus}(k)$$

Now, the current to be generated in the next sample being the reference value of current *i*_sc_^*^, duty cycle for the next sample is estimated as follows:

10 $${d}_{1}\left(k+1\right)= \frac{{V}_{sc}(k)}{{V}_{Bus}(k)}- \frac{{{L}_{sc}i}_{sc}^{*}}{{{t}_{s}V}_{Bus}\left(k\right)}+\frac{{L}_{sc}{i}_{sc}(k)}{{{t}_{s}V}_{Bus}\left(k\right)}$$

The differential equation governing the switching of battery-supercapacitor interface converter is given in (11), where *i*_*Bat*_ and *V*_*Bat*_ are the instantaneous current and voltage of Battery, *d*_*2*_ is the duty cycle of battery interface converter, *V*_*sc*_ is the supercapacitor bus voltage, *L*_*Bat*_ is the filter inductor in interface.

11 $${L}_{Bat}\frac{d{i}_{Bat}(t)}{dt}= {V}_{Bat}\left(t\right)- {d}_{2}(t){V}_{Bus}(t)$$

Discretizing the differential equation,

12 $${L}_{Bat}\frac{{i}_{Bat}\left(k+1\right)-{i}_{Bat}\left(k\right)}{{t}_{s}}= {V}_{Bat}\left(k\right)- {d}_{2}(t){V}_{Bus}(k)$$

Now, the current to be generated in the next sample is the reference value of current *i*_Bat_^*^, duty cycle for the next sample is estimated as follows:

13 $${d}_{2}\left(k+1\right)= \frac{{V}_{Bat}(k)}{{V}_{sc}(k)}- \frac{{{L}_{Bat}i}_{Bat}^{*}}{{{t}_{s}V}_{sc}\left(k\right)}+\frac{{L}_{Bat}{i}_{Bat}(k)}{{{t}_{s}V}_{sc}\left(k\right)}$$

#### SRM Converter dynamics

The switches G_1_ and G_2_ are turned ON as shown in Fig. 1, which results in + *V*_*Bus*_ voltage level at Phase A output terminals.

14 $${V}_{A}={V}_{Bus}=r {i}_{A}+\frac{d\varphi (\theta ,{i}_{A)}}{dt}$$

15 $${i}_{A}= {i}_{Bus}$$

The switches G_1_ and G_2_ are turned OFF, the complementary action of turned OFF G_1_ and G_2_ force diode D_1_ and D_2_ to turn ON, which results in *− V*_*Bus*_ voltage level at Phase A output terminals and the energy in phase A winding is freewheeled into source. During this interval,

16 $${V}_{A}=-{V}_{Bus}=r {i}_{A}+\frac{d\varphi (\theta ,{i}_{A)}}{dt}$$

17 $${i}_{A}=- {i}_{Bus}$$

Similar dynamics for other phases shall be provided as follows:

18 $${V}_{B}={V}_{Bus}=r {i}_{B}+\frac{d\varphi (\theta ,{i}_{B)}}{dt}$$

19 $${i}_{B}= {i}_{Bus}$$

during energizing phase B, and

20 $${V}_{B}=-{V}_{Bus}=r {i}_{B}+\frac{d\varphi (\theta ,{i}_{B)}}{dt}$$

21 $${i}_{B}=- {i}_{Bus}$$

during de-energizing phase B.

22 $${V}_{C}={V}_{Bus}=r {i}_{C}+\frac{d\varphi (\theta ,{i}_{C)}}{dt}$$

23 $${i}_{C}= {i}_{Bus}$$

during energizing phase C, and

24 $${V}_{C}=-{V}_{Bus}=r {i}_{C}+\frac{d\varphi (\theta ,{i}_{C)}}{dt}$$

25 $${i}_{C}=- {i}_{Bus}$$

during de-energizing phase C.

#### Dynamics of SRM:

The magnitude of the rotor flux space vector and its position are very important aspects in designing DTC. The rotational d-q coordinated system can easily be designed with the help of rotor magnetic flux space vector^60–62^. In many existing methods, the flux model has been implemented in this paper by utilizing monitored rotor speed and stator voltages along with currents. It is obtained from basic stationary reference frames (α, β) associated with the stator. The rotor flux space vector is achieved and are resolved into the α and β components as follows^63,64^.

26 $$\left[\left(1-\sigma \right){T}_{s}+{T}_{r}\right]\frac{d}{dt}{\varphi }_{r\alpha }=\frac{{L}_{m}}{{R}_{s}}{u}_{s\alpha }-{\varphi }_{r\alpha }-\omega {T}_{r}{\varphi }_{r\beta }-\sigma {L}_{m}{T}_{s}\frac{d}{dt}{i}_{s\alpha }$$

27 $$\left[\left(1-\sigma \right){T}_{s}+{T}_{r}\right]\frac{d}{dt}{\varphi }_{r\beta }=\frac{{L}_{m}}{{R}_{s}}{u}_{s\beta }-{\varphi }_{r\beta }-\omega {T}_{r}{\varphi }_{r\alpha }-\sigma {L}_{m}{T}_{s}\frac{d}{dt}{i}_{s\beta }$$

With ${T}_{r}=\frac{{L}_{r}}{{R}_{r}}$ *and *${T}_{s}=\frac{{L}_{s}}{{R}_{s}}$* and* $\sigma =1-\frac{{{L}_{m}}^{2}}{{L}_{s}{L}_{r}}$

Where *L*_*s*_ and *L*_*r*_* are* stator and rotor self-inductance, *L*_*m*_ is motor magnetizing inductance, *R*_*r*_* and R*_*s*_ are denoted for rotor and stator Resistance, ω is the angular speed of the rotor, *P*_*p*_ is pole pairs in SRM, ${T}_{r} is$ rotor time constant, ${T}_{s} is$ stator time constant, and $\sigma$ is used for leakage constant.

## Controller modelling

The control strategy of the proposed system is sophisticated and involves several interconnected layers, each serving specific purposes to ensure the efficient operation of the PV-assisted EV drive^65,66^. The first layer, which is akin to a pattern recognition machine learning algorithm, is responsible for setting the instantaneous torque based on the detected driving pattern, estimating the PV power output, and tracking the maximum available power from the PV system^67,68^. This layer relies on historical data and real-time inputs to make accurate predictions and optimize torque and power output. The second layer operates using mathematical models of the system and the motor itself. It employs these models to estimate the speed of the motor without relying on traditional speed sensors, thereby reducing cost and complexity. Additionally, it controls the hybrid power supply, adjusting the flow of power from the PV, battery, and supercapacitor to meet the instantaneous power demand of the drive^69,70^. The final layer is focused on coordinating the power flow throughout the entire interface. It ensures that power is distributed optimally among the different sources to maintain a stable DC bus voltage, regulate the system's response to load changes, and ensure efficient utilization of all available energy sources. This coordination is vital for the overall performance and reliability of the PV-assisted EV drive, as it ensures that the drive system operates efficiently and reliably under various operating conditions.

### Machine learning for torque and PV power estimation, MPP tracking

**Figure 2:**
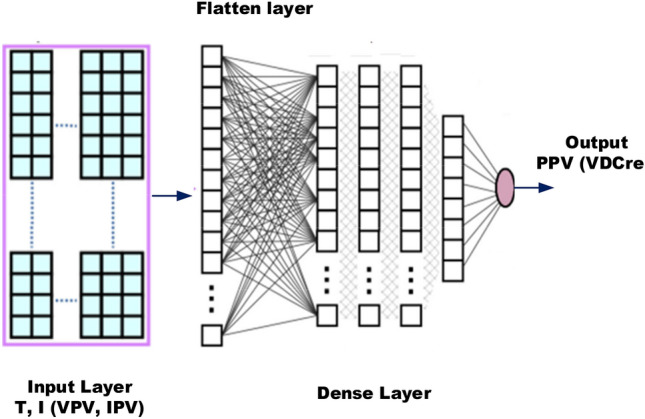
Multi-layered machine learning for pattern recognition for torque, PV power and MPP.

The machine learning algorithm in the proposed system is fed with three main types of input data: the difference between the actual motor speed and the reference speed for torque reference generation, the irradiance level for PV power estimation, and the error in the conductance for maximum power point (MPP) determination^71–73^. The algorithm employs a multi-layered approach, consisting of two inner layers, to establish a relationship between the input data and the desired output values. In the first inner layer, pattern recognition techniques are used to identify the appropriate torque reference, PV power level, or MPP reference. This process is illustrated in Fig. 2, which outlines the implementation of pattern recognition for each of these outputs. The structure of the machine learning model is carefully designed, and the weights associated with each connection between nodes are updated in each iteration based on a predetermined criterion. This iterative process allows the algorithm to learn and improve its performance over time, ultimately leading to more accurate torque references, PV power estimations, and MPP determinations.

**Figure 3:**
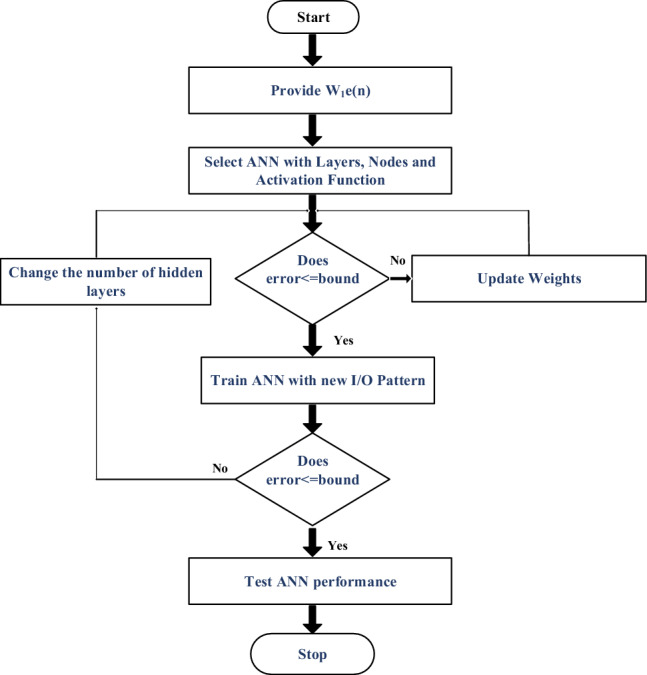
Algorithm for Multi-layered ML pattern recognition model implementation.

The pattern recognition-based machine learning algorithm utilized in this study incorporates a deep understanding of motor dynamics and solar irradiance variation to predict and optimize the electric vehicle's performance^74,75^. Specifically, the algorithm determines optimal torque settings based on input parameters like the error function of motor speed, reference speed, and irradiance for PV power estimation. In the initial layer, the algorithm estimates the required torque through a unique multi-layered machine learning model, which relies on deep neural networks. The model processes the input parameters to predict the output torque, taking into account the highly nonlinear characteristics of the electric vehicle's drive system. The training process employs an extensive dataset consisting of 14,000 samples. This dataset encompasses a wide range of driving scenarios, including various combinations of vehicle speeds, load profiles, and ambient conditions. The machine learning model undergoes iterative adjustments to its internal weights, improving its accuracy and predicting capability with each training cycle. The training process involves both forward and backward propagation techniques, refining the network's internal structure to enhance its performance^76,77^. This iterative learning process continues until the algorithm achieves a satisfactory level of accuracy in predicting the desired torque. The performance of the machine learning algorithm is evaluated through rigorous testing, ensuring its accuracy, precision, and robustness across diverse driving conditions. The algorithm's superior predictive capabilities are showcased through its ability to accurately determine torque references, enabling optimal power management and efficient energy utilization in light electric vehicles. These advancements in machine learning-based control algorithms not only enhance the efficiency and performance of electric vehicle drives but also pave the way for future innovations in autonomous driving and intelligent transportation systems. Algorithm for Multi-layered ML pattern recognition model implementation is shown in Fig. 3.

### Model based SRM Speed estimation

**Figure 4:**
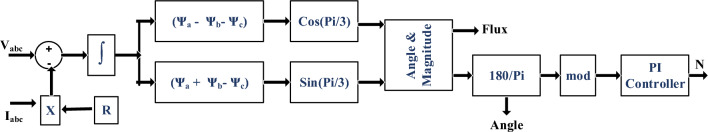
Speed estimation by MRAC.

Speed estimation is a critical aspect of motor control in electric vehicle (EV) systems. It is traditionally achieved through the use of speed sensors, which can be costly and introduce complexity to the system^78–80^. To address these challenges, we propose an innovative approach that leverages mathematical models and a model reference adaptive controller (MRAC) to estimate speed without the need for physical speed sensors. This approach is illustrated in Fig. 4, which shows a block diagram of the speed estimation process. In this system, the output of the switched reluctance motor (SRM) converter depends on both the voltage at the DC bus (*V*_*Bus*_) and the pulses generated by the pulse width modulation (PWM) generator. These converter voltages can be accurately estimated using mathematical expressions based on the motor and converter models. This eliminates the need for physical voltage sensors, significantly reducing the cost and complexity of the system. The core of the speed estimation process lies in the mathematical model of the SRM converter, which accurately describes the relationship between *V*_*Bus*_, the PWM pulses, and the motor speed. This model is utilized in the MRAC to adaptively estimate the motor speed based on the observed behavior of the converter. Overall, this approach offers a cost-effective and reliable alternative to traditional speed sensing methods, making it an attractive option for EV applications.

The following equations can estimate the speed:

28 $$v_{ds}^{s} = i_{ds}^{s} R_{s} + L_{ls} \frac{d}{dt}\left( {i_{ds}^{s} } \right) + \frac{d}{dt}\left( {\psi_{dm}^{s} } \right)$$

29 $$v_{ds}^{s} = \frac{{L_{m} }}{{L_{r} }}\frac{d}{dt}\left( {\psi_{dr}^{s} } \right) + \left( {R_{s} + \sigma L_{s} S} \right)i_{ds}^{s}$$

where $$\sigma = 1 - \frac{{L_{m}^{2} }}{{L_{r} L_{s} }}$$

30 $$\frac{d}{dt}\left( {\psi_{dr}^{s} } \right) = \frac{{L_{r} }}{{L_{m} }}v_{ds}^{s} - \frac{{L_{r} }}{{L_{m} }}\left( {R_{s} + \sigma L_{s} S} \right)i_{ds}^{s}$$

Similarly

31 $$\frac{d}{dt}\left( {\psi_{qr}^{s} } \right) = \frac{{L_{r} }}{{L_{m} }}v_{qs}^{s} - \frac{{L_{r} }}{{L_{m} }}\left( {R_{s} + \sigma L_{s} S} \right)i_{qs}^{s}$$

32 $$\frac{d}{dt}\left( {\psi_{dr}^{s} } \right) = \frac{{L_{m} }}{{T_{r} }}i_{ds}^{s} - \omega_{r} \psi_{qr}^{s} - \frac{1}{{T_{r} }}\psi_{dr}^{s}$$

33 $$\frac{d}{dt}\left( {\psi_{qr}^{s} } \right) = \frac{{L_{m} }}{{T_{r} }}i_{qs}^{s} + \omega_{r} \psi_{dr}^{s} - \frac{1}{{T_{r} }}\psi_{qr}^{s}$$

where $$T_{r} = \frac{{L_{r}^{{}} }}{{R_{r} }}$$.

Hence, the rotor speed is calculated by the below equation

34 $$\omega_{r} = \frac{d}{dt}\theta_{e} = \frac{1}{{\varphi_{r}^{2} }}\left\lfloor {\left( {\varphi^{s}_{dr } \frac{d}{dt}\varphi^{s}_{qr } - \varphi^{s}_{qr } \frac{d}{dt}\varphi^{s}_{dr } } \right) - \frac{{L_{m} }}{{T_{r} }}\left( {\varphi^{s}_{dr } i^{s}_{qs } - \varphi^{s}_{qr } i^{s}_{ds } } \right)} \right\rfloor$$

### Model based current control of HPS

**Figure 5:**
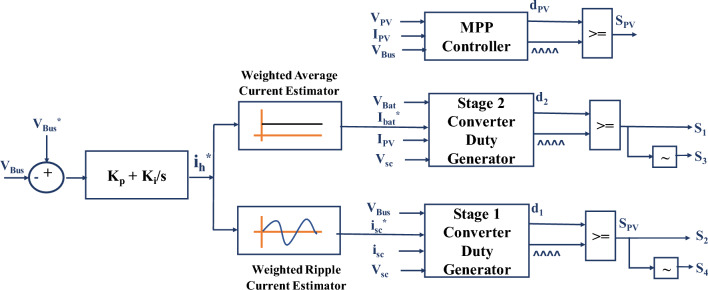
Model referred duty estimated PI current control for HPS.

The proposed control structure for the Hybrid Power Supply (HPS) system in Light Electric Vehicles (LEVs) is a novel approach that combines principles of Proportional-Integral (PI) control for current reference generation and Model Reference Adaptive Controller (MRAC) for duty cycle generation. The main objectives of this control algorithm are to regulate the DC bus voltage to its permissible value and facilitate instantaneous power supply sharing between the battery and supercapacitor for varying load conditions. The control scheme, as depicted in Fig. 5, consists of two primary components: the current reference generation and the duty cycle generation. The first part focuses on generating the appropriate current references for the battery and supercapacitor based on the desired DC bus voltage. It involves the use of a PI controller that adjusts the current references to maintain the DC bus voltage within acceptable limits. The second part of the control scheme involves the generation of the duty cycles for the converters that interface with the battery and supercapacitor. These duty cycles are calculated based on the power sharing requirements and the load variations. The MRAC plays a crucial role in ensuring that the duty cycles are adjusted in real-time to meet the dynamic power demands of the system. Overall, the proposed control structure offers a robust and efficient solution for regulating the HPS system in LEVs. It provides precise control over the DC bus voltage and enables seamless power sharing between the battery and supercapacitor.

The PV interface converter, situated within the Hybrid Power Supply (HPS) system of Light Electric Vehicles (LEVs), performs a critical role in managing power distribution efficiently. It operates independently from the battery and supercapacitor converters, ensuring that the Direct Current (DC) bus receives the maximum available power from the solar panels at all times. This autonomous operation ensures the optimal utilization of solar energy in the system. Meanwhile, the battery and supercapacitor converters complement the power supply by providing additional power when the PV system alone cannot meet the demand. The battery and supercapacitor converters are designed to distribute the remaining power needed to meet the load demand equitably. This ensures a balanced and consistent power supply to the vehicle. To facilitate seamless power distribution among the PV, battery, and supercapacitor converters, a sophisticated control scheme has been developed. This control strategy is based on a model-referred duty estimation-based Proportional-Integral (PI) current regulation approach. This approach continually assesses the current states and references of the converters to generate optimized switching pulses. These pulses regulate power flow, maintain the DC bus voltage, and enable effective power sharing among the converters. As a result, the model-referred duty estimation-based PI current regulation scheme ensures efficient and balanced power distribution within the HPS system of LEVs. This innovative approach significantly contributes to the advancement of sustainable and eco-friendly electric transportation by improving vehicle performance, reliability, and energy efficiency.

Error in the DC bus voltage serves as input to PI regulator, which determines the magnitude and direction of current supplied by the hybrid combination of battery and super capacitor. Then, the weighted average current estimator separates the reference for battery current and weighted transient current estimator separates the reference current to be absorbed or delivered by supercapacitor at instant. The weights for average and ripple current estimators are the factors by which hybrid reference current *i*_h_^*^ is raised by (1-*d*_*2nom*_) and (1-*d*_*1nom*_) respectively, where *d*_*2nom*_ and *d*_*1nom*_ are the nominal duty cycles of stage 1 interface converter and stage 2 interface converter respectively. During average and ripple extraction from reference current, the averaging of reference current is limited by the c-rate of battery and the ripple extracted shall be supplied by the supercapacitor instantaneously. Further, the model equations described in (6) concerning the condition satisfied in (7) generate the instantaneous duty cycle for PV interface converter. The duty thus generated is compared to constant frequency triangular carrier waveform to generate switching pulses for S_PV_. Also, Eq. (16) serves as a reference to generate duty cycle for the stage 2 converter while Eq. (10) serves as a reference for duty cycle generation for stage 1.

### Coordinated control of drive

**Figure 6:**
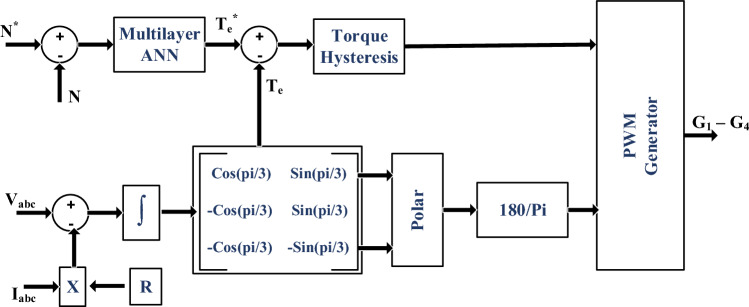
Control block diagram for SRM.

**Figure 7:**
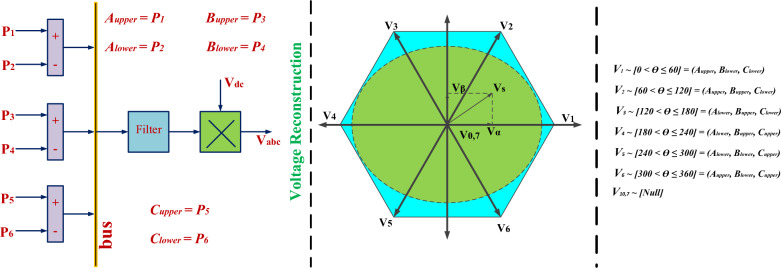
Vector based instantaneous switch combinations for SRM current control.

Coordinated control for optimal current regulation into Switched Reluctance Motor (SRM) for speed and torque commands plays a crucial role in ensuring the smooth and efficient operation of the SRM drive^81–83^. The control scheme is depicted in Fig. 6, where the SRM model estimates torque based on phase voltages and currents. The obtained instantaneous torque reference from the supervised model is then compared with the estimated torque, resulting in torque hysteresis. Similarly, flux hysteresis is developed from the SRM model, as illustrated in Fig. 6. These two hysteresis components serve as inputs for determining the instantaneous voltage vector, as presented in Table 1. In Fig. 7, the corresponding voltage vectors are generated from the integration of the estimated speed to identify the sector. However, due to the specific topology of the converter with four switches, one switch common in all three phases, the generated vectors are identified differently, as shown in Fig. 7. Accordingly, the corresponding switches of the leg are turned ON to control the current flow into the SRM. This comprehensive approach ensures the effective and coordinated control of the SRM drive, optimizing its performance and efficiency in various operating conditions.

**Table 1:** Current Control Space Vector Dynamic Switching.

Flux error	Torque error	Magnitude shift	Phase shift
Positive	Positive	Increase	Anti-clockwise
Positive	Negative	Increase	Clockwise
Negative	Positive	Decrease	Anti-clockwise
Negative	Negative	Decrease	Clockwise

## Simulation results and performance evaluation

A detailed simulation of the proposed drive was conducted using MATLAB/SIMULINK, wherein the load was modeled to reflect real-world electric vehicle drive cycles, encompassing scenarios like acceleration, maintaining a constant velocity, and vehicle deceleration. The parameters employed in the system simulation are outlined in Table 2, covering various aspects such as power sources, the motor itself, power switches, filter elements, and specifications pertinent to machine learning. Throughout the simulation, the proposed drive underwent rigorous testing to assess its performance across a spectrum of critical metrics. Initially, the accuracy and real-time viability of the machine learning algorithm were scrutinized for its capacity to generate torque references, estimate PV power, and identify the maximum power point (MPP) voltage. Subsequently, the regulation of hybrid power supplies (HPs) and the distribution of power among diverse sources were evaluated. Furthermore, the drive was put through a battery of tests to evaluate its response in different operational scenarios. This encompassed examining steady-state torque ripple, the transient response of torque and speed, and the response when reversing the speed command. Through this exhaustive testing, the performance characteristics and the efficacy of the proposed drive were gauged, ensuring a thorough understanding of its capabilities and limitations in varied operating conditions.

**Table 2:** Simulation Parameters.

Parameter	Simulation
PV source	660 W_p_, V_OC_ = 36 V, I_SC_ = 5 A
Battery source	200 AH, I_max_ = 20 A, V = 12 V
Super capacitor	58 F, 18 V
DC Bus nominal voltage	48 V
L_Bat_	0.8 mH
L_Bat_	1 mH
L_sc_	1 mH
C_DC_	440 µF
K_p_, K_i_	0.0325, 0.224
SRM power rating	5 HP
Nominal speed	3000 rpm
Maximum current	20 A
Supervised learning parameters	Inputs: e(N) (or I or ΔG)
	Outputs: T* (or P_PV_ or V_PV_*)
	Error bound: 0.01
	Activation Function: Sigmoid

### Performance of supervised learning

**Figure 8:**
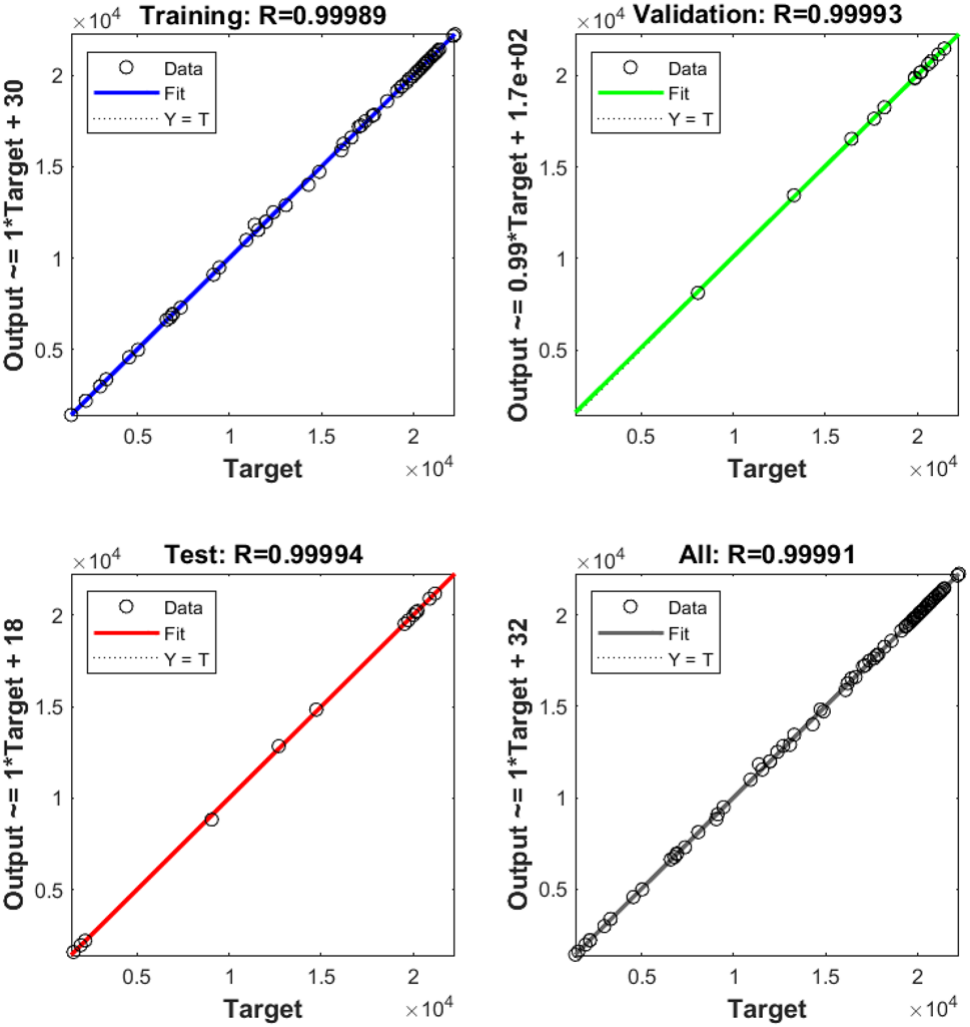
Training of machine.

**Figure 9:**
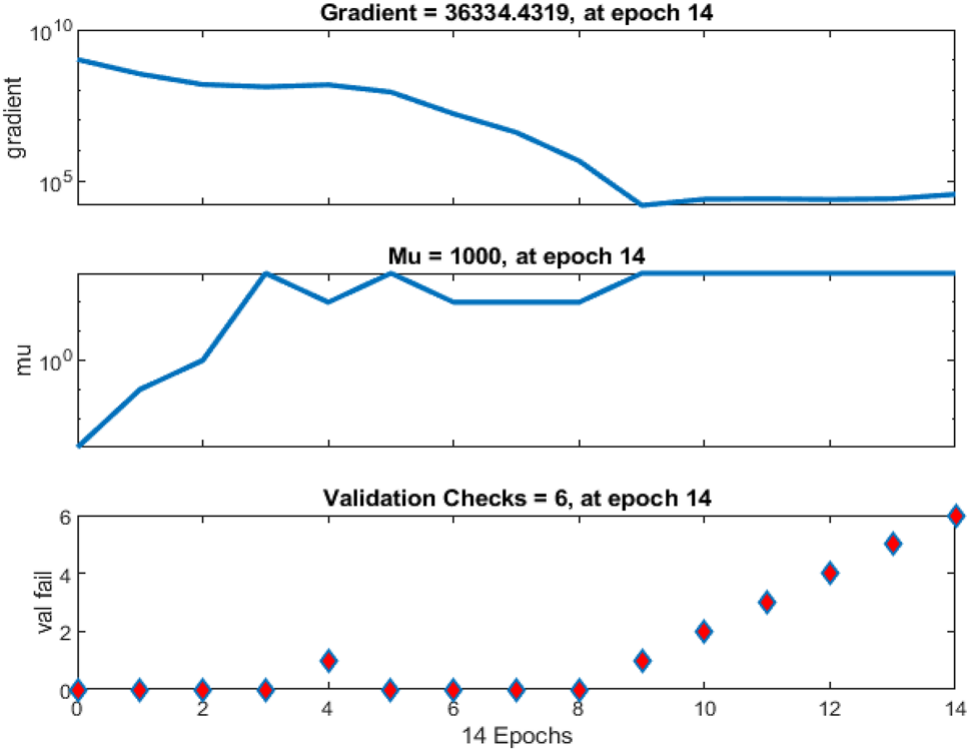
Gradient of mean squared error and validation checks.

**Figure 10:**
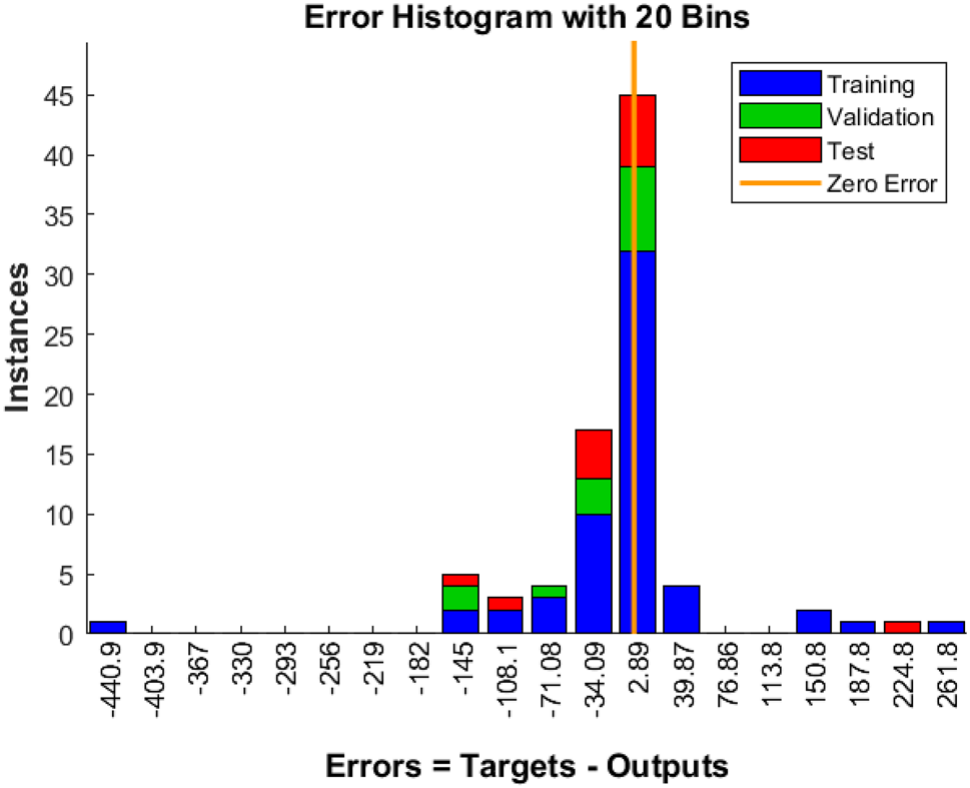
Error histogram for twenty test samples.

**Figure 11:**
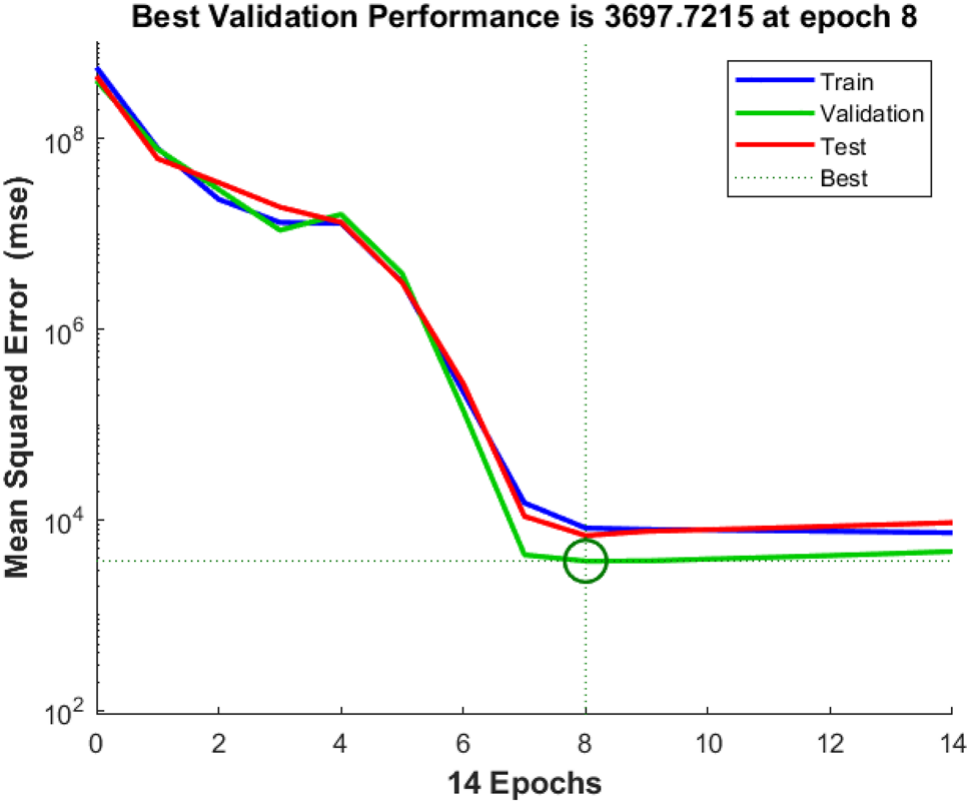
Performance of supervised learning pattern.

The training and validation processes of the machine learning algorithm were meticulously monitored and evaluated. Table 3 elucidates the sample combinations for training data, delineating the pairing of torque reference with corresponding speed and PV power estimations. The subsequent analyses of training and validation performance are captured through various figures. For instance, Fig. 8 offers insight into the tracking of target values across iteration cycles, with each iteration cycle manifesting a distinct fitness level denoting the learning capability of the artificial neural network (ANN) for the training pattern^84,85^. Moreover, Fig. 9 illustrates the gradient of error, showcasing how it stabilizes after eight iterations. The validation checks are also graphically represented in Fig. 9. Figure 10 presents an error histogram for a sample set of twenty data points, exhibiting the frequency distribution of errors encountered during the validation process. Encouragingly, for 95 percent of these data points, the mean square error was observed to be within a negligible range of 0.1 percent. To further validate the efficacy of the algorithm, Fig. 11 offers an in-depth analysis of the mean squared error, with specific emphasis placed on the zeroing of mean squared error from the eighth iteration onwards. This meticulous analysis of training and validation processes serves to affirm the reliability and robustness of the developed machine learning algorithm in accurately estimating torque reference, speed, and PV power.

**Table 3:** Sample Training Data for ANN.

Speed error (rpm)	Change in torque reference (N-m)	Irradiance (W/m^2^)	Normalized power generated (percentage)
22	− 0.2	504	25.39472593
29	− 0.28	465	23.13561481
98	− 0.9	465	23.13561481
90	− 0.82	487	24.40998519
− 42	0.4	569	28.92241481
− 40	0.39	505	25.21515556
− 38	0.37	474	23.41945185

### Performance of HPS

**Figure 12:**
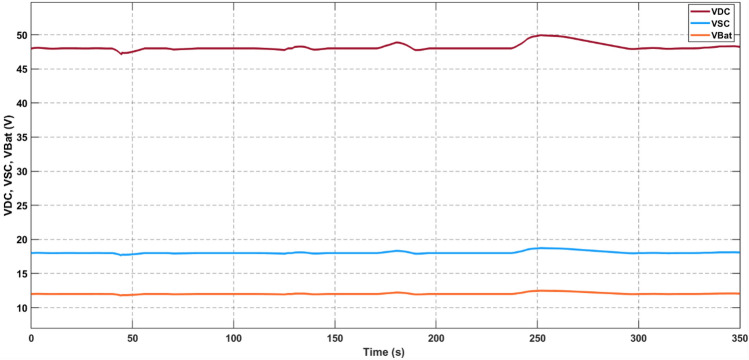
Voltage of DC Bus, Supercapacitor and Battery bank.

In Fig. 12, the voltage profiles of the DC bus, supercapacitor bus, and battery bank are depicted, demonstrating their adept regulation to nominal values with precision, as demonstrated in Fig. 6. A comprehensive examination of this regulation process and its associated voltage stress is provided in the subsequent subsection. Notably, the ensuing discussion reveals an admirably stringent regulation standard, wherein a deviation of under 5 percent is observed across the entire span of load variations within the nominal range.

**Figure 13:**
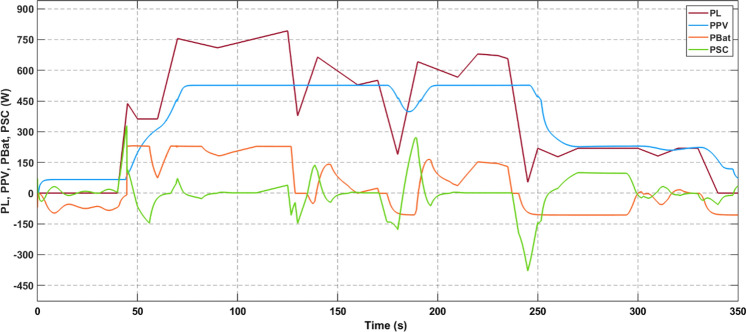
Power delivered by sources and load power demand.

In the simulated scenario, the voltage regulation is meticulously maintained to nominal values, ensuring precise control over the distribution of power among the sources. As displayed in Fig. 13, the power shares reflect the current allotments among the different components. Furthermore, the figure visually represents how the PV-generated power, which is dependent on irradiance, is channeled to the DC bus. Meanwhile, the battery and supercapacitor share the remaining power requirements, with the supercapacitor rapidly accommodating any sudden load variations. This flexible arrangement ensures the efficient and seamless adaptation of the system to changing conditions, optimizing the performance of the light electric vehicle under different driving scenarios.

### Performance of SRM control

**Figure 14:**
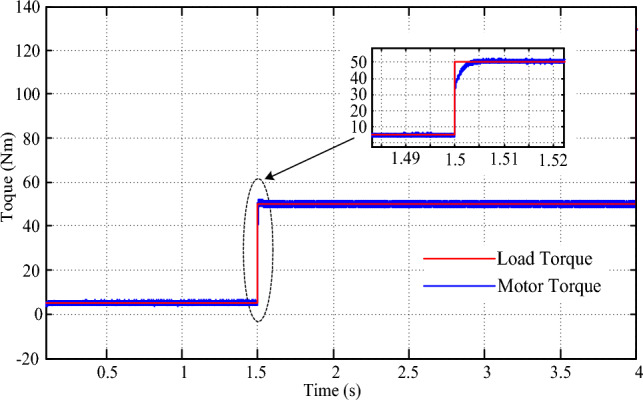
Torque response for step change in torque command.

**Figure 15:**
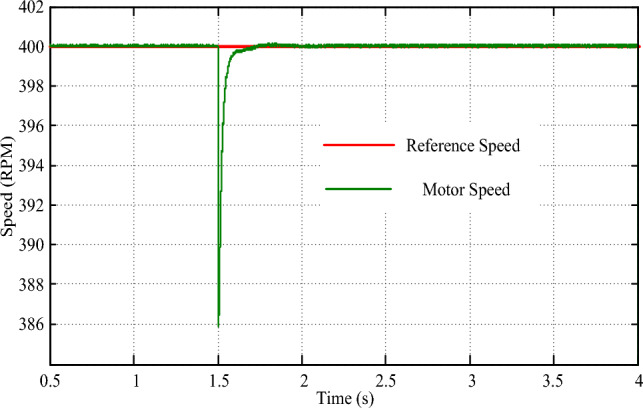
Speed response for step change in torque command.

The performance of the drive in response to a 50 N-m torque increase was examined through simulation. As shown in Fig. 14, the drive torque response exhibits precise tracking of the new torque demand, with a transient time of just 0.01 s and zero steady-state error. This rapid adjustment is complemented by a minor dip of 15 rpm in speed, as depicted in Fig. 15, which is quickly resolved within 0.4 s of the changeover. These results underscore the drive's ability to efficiently adapt to abrupt variations in torque demand, ensuring a smooth and uninterrupted driving experience. The implementation of a multi-layered machine learning algorithm, including pattern recognition for instantaneous torque setting and PV power estimation, contributes significantly to the drive's agility and accuracy in responding to dynamic torque demands.

**Figure 16:**
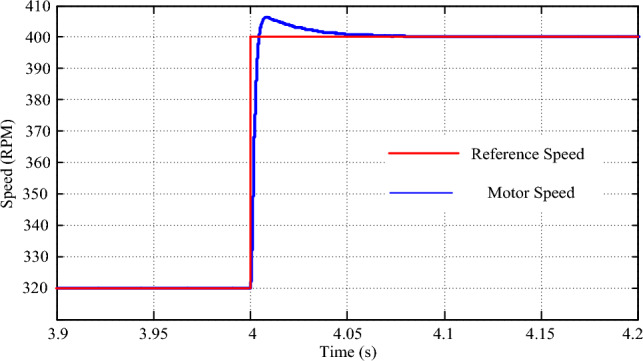
Speed response for a step change in reference speed.

**Figure 17:**
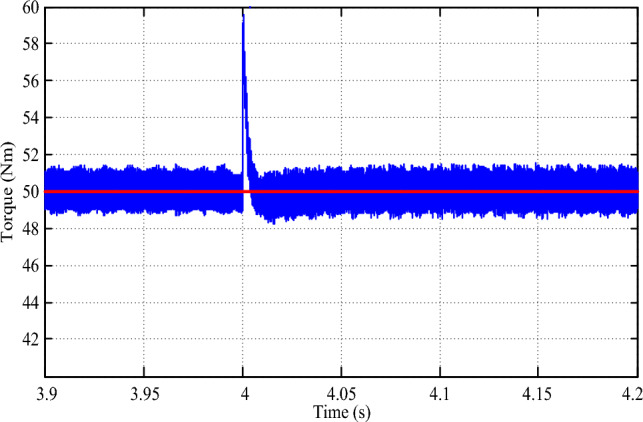
Torque response for a step change in reference speed.

Simulating the drive for an 80 N-m step change in speed demand provides further insights into its robust performance. As illustrated in Fig. 16, the drive speed precisely tracks the new speed demand, exhibiting zero steady-state error and a transient time of merely 0.08 s. Concurrently, a surge of 10 N-m in drive torque is observed in Fig. 17 during the transition, swiftly settling within 0.01 s. The torque response under these conditions highlights the drive's effective management of sudden changes in speed demand, showcasing its adaptability and reliability in varying driving scenarios. The implementation of the multi-layered machine learning algorithm significantly contributes to this precise and agile response, underscoring its role in ensuring smooth and consistent drive performance.

**Figure 18:**
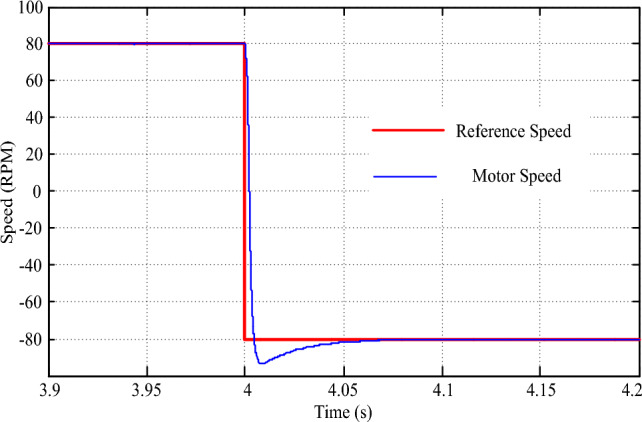
Speed response for speed reversal command.

**Figure 19:**
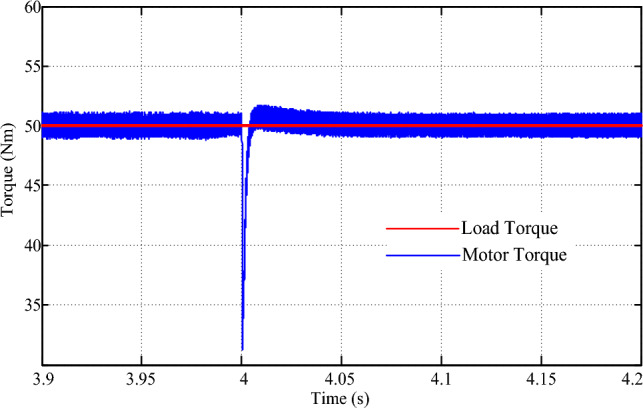
Torque response for reversal of speed.

Simulating a scenario of a sudden speed reversal from + 80 rpm to – 80 rpm provides crucial insights into the drive's resilience and performance under extreme conditions. In this experiment, we examined how the drive responds to such abrupt changes in speed demand, ensuring the safety and stability of the vehicle in unpredictable situations. As depicted in Fig. 18, the drive's speed tracking capabilities are commendable, showcasing an error-free transition and a remarkably swift transient time of just 0.08 s. This rapid response underscores the drive's agility and adaptability, vital attributes for navigating dynamic and ever-changing environments. However, the transition also exposes a brief dip in drive torque, as illustrated in Fig. 19. This temporary dip occurs due to the absence of a load during the transient speed reversal, but it is rapidly corrected within a mere 0.01 s. This quick recovery reflects the drive's robustness and its ability to maintain consistent performance even during the most challenging conditions. By simulating scenarios such as these, we can better understand the drive's capabilities and potential areas for improvement. Furthermore, it allows us to refine control strategies and drive algorithms, ultimately enhancing the overall performance, safety, and efficiency of electric vehicles.

### Comparison to existing power supplies

To provide a comprehensive evaluation of the proposed hybrid power supply (HPS) system and its accompanying control system, we conducted a rigorous comparison with existing power supplies commonly used in PV-assisted electric vehicle (EV) drives. This comparison aimed to assess the robustness and accuracy of the proposed HPS and control mechanism across various performance metrics, including DC bus regulation, stress on the supercapacitor for transient requirements, and optimal sizing of power supply components. Table 4 serves as a visual representation of the comparative analysis, highlighting the key attributes and performance characteristics of the proposed HPS and control system. It illustrates how the proposed system fares against conventional power supplies in terms of addressing transient load demands, maintaining the stability of the DC bus voltage, and ensuring the overall reliability and efficiency of the power delivery. Through this comprehensive comparison, we aim to demonstrate the superiority of the proposed HPS and control mechanism in terms of robustness, accuracy, and performance, setting a new standard for PV-assisted EV drives (Table 5).

**Table 4:** Comparison of Power Supplies for PV assisted EV Drive.

	^17^	^21^	^25^	Proposed control for conventional converters	Proposed control for cascaded converters
Power rating	480 W	40 W	80 W	900 W	900 W
DC bus voltage	48 V	48 V	48 V	48 V	48 V
DC bus regulation	12.5%	5.3%	4.25%	3.125%	2.7%
Super capacitor ratings	65 F, 18 V	65 F, 18 V	65 F, 18 V	58 F, 18 V	58 F, 18 V
Voltage stress percentage	2.5%	2.4%	2.4%	2.2%	1.6%

**Table 5:** Power Converters sizing comparison.

	Conventional drive	Proposed drive
HPS stage 2 inductor size	8 mH	0.56 mH
HPS stage 2 series switch voltage stress	1 pu	0.16 pu
SRM converter power switches	6	4

### Drive component sizing comparison

The merit of the proposed HPS topology in terms of steady-state ripple in battery interface inductor and series switch voltage stress is evaluated in this section. The mathematical expression for inductor current ripple is obtained as follows:

For a bi-directional converter with inductor at battery side for conventional topology, following the differential equation as

35 $${L}_{Bat}= \frac{{V}_{Bus}({V}_{Bus}-{V}_{Bat})}{{\Delta i}_{LBat}{f}_{s}{V}_{Bat}}$$

And for cascade converter topology, following the differential equation as in (14)

36 $${L}_{Bat}= \frac{{V}_{sc}({V}_{sc}-{V}_{bat})}{{\Delta i}_{LBat}{f}_{s}{V}_{Bat}}$$

Substituting for considered nominal values for ${V}_{Bus}$, ${V}_{Bat}$, and ${V}_{sc}$, the following expressions for battery inductor size are obtained for conventional topology as

37 $${L}_{Bat}= \frac{144}{{\Delta i}_{LBat}{f}_{s}}$$

And for cascaded converter topology it is

38 $${L}_{Bat}= \frac{9}{{\Delta i}_{LBat}{f}_{s}}$$

The percentage change in battery inductor size as per considered nominal values of voltages is obtained as

$${\% \Delta L}_{Bat}=93.75$$

Now, the voltage sizing of diodes and switches in SRM converter was obtained from the blocking voltage level during turn OFF interval of the respective switch or diode. In these intervals, the blocking voltage across the switch combination was obtained as V_sw_ = V_S_/2. The diode during turned OFF, should block the maximum value of source voltage. Therefore, the voltage rating of any diode was V_D_ = V_S_/2. The RMS current rating of power switches is determined from power to be delivered by the converter. Now, the RMS current rating of G_x_ or D_x_ where X = 2,3,4 is obtained as

39 $${I}_{rms,X}= \frac{{P}_{avg}}{3{V}_{ph,rms}}$$

and that of G_1_ and D_1_ is 3I_rms,X_

The battery interface converter, a critical component in electric vehicles (EVs) using photovoltaic (PV) power, was subjected to rigorous analysis in this study. A comparison was made between the conventional topology and the proposed cascaded converter topology, focusing on the reduction of component sizes while maintaining or improving performance. The battery interface inductor, an essential element, was computed using Eqs. (19) and (20) for both topologies. It was found that the proposed cascaded converter topology led to a substantial reduction in the inductor's size. Additionally, the voltage stress on series switch S2 was evaluated under OFF conditions. The results showed a significant decrease in voltage stress from 1 pu in the conventional topology to only 0.16 pu in the cascaded converter topology. This reduction in voltage stress, along with the downsizing of the battery interface components, is a testament to the effectiveness of the proposed topology. Furthermore, the sizing of switches in the switched reluctance motor (SRM) converter was optimized, resulting in fewer switches and improved efficiency without compromising performance. The results of this comparative analysis underscore the potential of the proposed topology to enhance the performance and efficiency of battery interface converters in EVs using PV power.

### Comparison to existing drive output characteristics

The performance of the proposed hybrid power supply (HPS) with the proposed control scheme was compared to existing power supplies typically used in photovoltaic (PV)-assisted electric vehicle (EV) drives. Additionally, the proposed control strategy was also applied to a conventional power supply to assess its robustness and effectiveness. Table 6 provides a detailed comparison of the proposed control scheme with the HPS in terms of DC bus regulation, supercapacitor stress for transient requirements, and power supply component sizing. The results demonstrate the robustness and accuracy of the proposed control strategy, particularly when used in conjunction with the HPS. The analysis indicates that the proposed control scheme can effectively regulate the DC bus voltage, manage transient requirements without placing excessive stress on the supercapacitor, and optimize power supply component sizing. These findings underscore the potential of the proposed control scheme and HPS in enhancing the performance and efficiency of PV-assisted EV drives.

**Table 6:** Comparison of Power Supplies for PV-assisted EV Drive.

	^48^	^52^	^53^	^54^	Proposed drive
Torque ripple	0.06 pu	0.06 pu	0.05 pu	0.05 pu	0.04 pu
Torque settling	0.02 s	0.02 s	0.02 s	0.015 s	0.01 s
Speed settling	0.8 s	0.8 s	0.8 s	0.6 s	0.5 s
Speed reversal	0.8 s	0.8 s	0.8 s	0.6 s	0.7 s

## Conclusion and future research directions

In conclusion, this paper has presented a comprehensive study on the development and performance evaluation of a novel PV-assisted EV drive system with a focus on efficient and sustainable power management. We introduced a unique topology and mathematical model for the proposed drive, which integrates hybrid energy storage solutions and advanced control strategies, including machine learning. Our simulation results demonstrate the effectiveness and real-time feasibility of the machine learning algorithm for torque reference generation, PV power estimation, and MPP voltage identification, with a mean squared error within 0.1 percent for 95 percent of samples after the eighth iteration. Additionally, we showcased the robustness and accuracy of our control scheme through various performance indices such as DC bus regulation, power sharing among various sources, and transient response, with stringent regulation of less than 5 percent observed for all possible variations in the nominal range of the load. Our study also introduced a new approach to current control in a hybrid power system that addresses load changes effectively and efficiently. This approach, based on model reference adaptive control, offers improved performance over traditional methods. Additionally, our proposed control scheme for the SRM drive provides precise torque control, reduced torque ripple, and fast transient response. Our simulation results confirm that our proposed control strategy successfully handles changes in torque demand and speed commands, ensuring accurate and rapid responses, with a torque ripple of 0.04 pu and a speed settling time of 0.5 s for a step change in reference speed. We compared the performance of our proposed HPS with existing power supplies for PV-assisted EV drives, showcasing superior DC bus regulation and reduced supercapacitor voltage stress, with a DC bus regulation as low as 2.7 percent and a supercapacitor voltage stress as low as 1.6 percent. Moreover, we presented a detailed analysis of the sizing of drive components, including the battery interface inductor and series switch, demonstrating significant reductions in size and voltage stress with our proposed topology, with a 93.75 percent reduction in battery interface inductor size and a 0.16 pu series switch voltage stress.

Overall, this study makes several significant contributions to the field of PV-assisted EV drives. We introduce a novel topology and mathematical model, propose efficient control strategies, and provide detailed simulations and analyses of the performance of the proposed system. Our work demonstrates the feasibility and benefits of integrating PV, battery, and supercapacitor energy storage systems in an EV drive, paving the way for more sustainable and efficient electric mobility solutions. Furthermore, our findings contribute to the development of advanced control and power management strategies for renewable energy-based transportation systems, promoting the adoption of PV-assisted EV drives and supporting the transition towards a greener and more sustainable future.

Future research directions for PV-assisted EV drives encompass several key areas. One such area is the advancement of control strategies. Deep reinforcement learning and artificial intelligence have shown promise in enabling real-time optimization of PV-assisted EV drives. Research in this domain can lead to more sophisticated and adaptive control algorithms that optimize energy efficiency and overall performance. Another area of interest is the exploration of advanced multi-level converter designs. These converters have the potential to improve power density and reduce component stress, thereby enhancing the overall efficiency and reliability of PV-assisted EV drives. Innovative battery management techniques also offer promising avenues for future research. Energy storage integration is critical for the effective operation of PV-assisted EV drives, and developing novel battery management systems can improve the overall energy efficiency and lifespan of these systems. Continuous system optimization and performance evaluation are also important areas for future research. By rigorously evaluating the performance of PV-assisted EV drives under various operating conditions, researchers can identify areas for improvement and fine-tune the design and control strategies to enhance the system's reliability and efficiency. Furthermore, researchers can extend the scope of their work to include other renewable energy sources for hybrid energy systems. This can involve integrating technologies such as wind power or geothermal energy to create more robust and resilient energy systems for EVs. Rigorous real-world testing and validation are crucial for ensuring the reliability and safety of PV-assisted EV drives. Researchers should collaborate with industry partners and government agencies to conduct extensive testing and validation under various operating conditions to ensure that these systems meet the highest standards of safety and performance. Finally, accelerating the commercialization and adoption of PV-assisted EV drives is essential for realizing their full potential. This can be achieved through industry-government partnerships and incentives that encourage the widespread adoption of these systems. By focusing on these key areas, researchers can help advance the state of the art in PV-assisted EV drives and contribute to a more sustainable and resilient future in the realm of electric mobility.

## Data Availability

The datasets used and/or analysed during the current study available from the corresponding author upon reasonable request.
